# Association of pain and risk of falls in community-dwelling adults: a prospective study in the Survey of Health, Ageing and Retirement in Europe (SHARE)

**DOI:** 10.1007/s41999-022-00699-1

**Published:** 2022-10-13

**Authors:** Giulia Ogliari, Jesper Ryg, Karen Andersen-Ranberg, Lasse Lybecker Scheel-Hincke, Jemima T. Collins, Alison Cowley, Claudio Di Lorito, Louise Howe, Katie R. Robinson, Vicky Booth, David A. Walsh, John R. F. Gladman, Rowan H. Harwood, Tahir Masud

**Affiliations:** 1 Department of Health Care for Older People (HCOP), Queen’s Medical Centre, Nottingham University Hospitals NHS Trust, Nottingham, Nottinghamshire UK; 2 NIHR Nottingham Biomedical Research Centre, Nottingham, UK; 3 Department of Geriatric Medicine, Odense University Hospital, Odense, Denmark; 4 Geriatric Research Unit, Department of Clinical Research, University of Southern Denmark, Odense, Denmark; 5 Unit for Epidemiology, Biostatistics and Biodemography, Department of Public Health, University of Southern Denmark, 5000 Odense, Denmark; 6 Centre for Rehabilitation and Ageing Research, Academic Unit of Injury, Inflammation and Recovery Sciences, School of Medicine, University of Nottingham, Nottingham, UK; 7 University Hospitals of Derby and Burton NHS Foundation Trust, Derby, UK; 8 Research and Innovation, Nottingham University Hospitals NHS Trust, Nottingham, UK; 9 Pain Centre Versus Arthritis, University of Nottingham, Nottingham, UK; 10 Sherwood Forest Hospitals NHS Foundation Trust, Sutton-in-Ashfield, UK; 11 NIHR Applied Research Collaboration—East Midlands, Nottingham, UK; 12 School of Health Sciences, University of Nottingham, Nottingham, UK

**Keywords:** Pain, Falls, Population-based prospective study, Joint pain, Multisite pain, Ageing

## Abstract

**Purpose:**

To investigate the longitudinal associations between pain and falls risks in adults.

**Methods:**

Prospective cohort study on data from 40,636 community-dwelling adults ≥ 50 years assessed in Wave 5 and 6 in the Survey of Health, Ageing and Retirement in Europe (SHARE). Socio-demographic and clinical information was collected at baseline (Wave 5). At 2-year follow-up (Wave 6), falls in the previous 6 months were recorded. The longitudinal associations between pain intensity, number of pain sites and pain in specific anatomic sites, respectively, and falls risk were analysed by binary logistic regression models; odds ratios (95% confidence intervals) were calculated. All analyses were adjusted for socio-demographic and clinical factors and stratified by sex.

**Results:**

Mean age was 65.8 years (standard deviation 9.3; range 50–103); 22,486 (55.3%) participants were women. At follow-up, 2805 (6.9%) participants reported fall(s) in the previous 6 months. After adjustment, participants with moderate and severe pain at baseline had an increased falls risk at follow-up of 1.35 (1.21–1.51) and 1.52 (1.31–1.75), respectively, compared to those without pain (both *p* < 0.001); mild pain was not associated with falls risk. Associations between pain intensity and falls risk were greater at younger age (*p* for interaction < 0.001). Among participants with pain, pain in ≥ 2 sites or all over (multisite pain) was associated with an increased falls risk of 1.29 (1.14–1.45) compared to pain in one site (*p* < 0.001).

**Conclusions:**

Moderate, severe and multisite pain were associated with an increased risk of subsequent falls in adults.

**Supplementary Information:**

The online version contains supplementary material available at 10.1007/s41999-022-00699-1.

## Key Summary Points

**Aim:**

To explore the longitudinal associations between pain characteristics at baseline and subsequent falls risks, at 2-year follow-up, in community-dwelling adults aged ≥ 50 years, in the Survey of Health, Ageing and Retirement in Europe (SHARE).

**Findings:**

Higher intensity of pain and number of pain sites at baseline were associated with an increased risk of subsequent falls in community-dwelling adults, in a dose–response way, independent of socio-demographic and clinical characteristics. The strength of the association between pain intensity and falls risk varied by age, being greater in middle-aged adults.

**Message:**

The association between pain intensity and falls risk is of greater clinical significance in middle-aged adults versus older adults.

**Supplementary Information:**

The online version contains supplementary material available at 10.1007/s41999-022-00699-1.

## Introduction

Falls are a major public health issue [1, 2]. One in three older adults falls in a year and falls lead to serious injuries in about 5–15% of cases [3–6]. Women have a higher risk of falls and fall-related injuries than men [7]. Falls have been associated with increased risks of morbidity, mortality, institutionalisation and healthcare costs [8]. Established risk factors for falls include older age, female sex, living alone, self-rated health, co-morbidities, certain medications, vision and hearing impairment and previous falls [9]. Obesity and underweight have been associated with a greater falls risk, compared to normal weight [10, 11]. Physical inactivity and poorer muscle strength may contribute to a steeper decline in mobility and, as a result, to falls risk [12, 13]. To prevent falls, we need to identify falls risk factors that are highly prevalent and potentially reversible [2].

Pain is highly prevalent in adults, with prevalence estimates varying by age and sex [14]. About half of older adults in the U.S. reported pain in at least one part of the body in the previous month [15]. In the UK, between one-third and one-half of adults reported chronic pain [16]. Women reported pain more frequently than men [14–17]. Pain has been associated with greater falls risk [18, 19]. However, it is unclear whether pain is associated with falls independently of co-variates. Pain is more common in adults with co-morbidities that are known falls risk factors, such as arthritis, obesity and depression [15]. However, pain could be causally linked to falls, by affecting physical activity and muscle strength. In older adults, chronic pain may lead to physical inactivity [20]. Pain has also been associated with decreased muscle strength, slower gait speed and worse lower extremity function and poorer balance in older adults [15, 21]. Furthermore, pain could directly contribute to falls risk by drawing attention away from mobility tasks [22].

It is unclear how age may modulate the associations between pain and falls and whether these may differ in middle-aged adults compared to older adults [23, 24]. In older adults, risk factors for falls other than pain—such as sensory impairments, co-morbidities and use of medications [9]—are more prevalent and may contribute to falls risk more than does pain. In contrast, in middle-aged adults, pain may be a significant falls risk factor due to the absence or lower prevalence of other falls risk factors (i.e., less competing risk factors for falls). Furthermore, the associations between pain in specific anatomic sites (e.g., knee pain) and falls risk need to be further elucidated [18].

We explored the longitudinal associations between: (1) intensity of pain; (2) number of pain sites and (3) presence of pain at specific anatomic sites, respectively, at baseline, and subsequent falls risk at follow-up in community-dwelling adults aged ≥ 50 years in the Survey of Health, Ageing and Retirement in Europe (SHARE). Further, we examined whether the association between intensity of pain and falls risk varied by age.

We hypothesised that: (1) greater intensity of pain; (2) pain in ≥ 2 sites or all over (multisite pain) versus pain in one site or no pain and (3) pain versus no pain in specific anatomic sites at baseline would be associated with an increased risk of subsequent falls. We also hypothesised that the strength of the association between intensity of pain and falls risk would vary across age groups, being greater in younger age.

## Methods

### Study design and population

SHARE is a longitudinal survey of ageing processes in individuals in European countries and Israel, as previously described [25–27]. Nationally representative samples from different countries were based on probability household samples; eligible households had at least one non-institutionalised member aged ≥ 50 years who spoke the official language of the country and was not living abroad at the time of survey. Eligible participants were individuals aged ≥ 50 years and their partners, irrespective of age. They were followed over time and refreshment samples of new individuals were enrolled in new waves to compensate for dropout. The first wave of data collection was conducted in 2004, followed by biennial survey waves. Trained interviewers conducted standardised computer assisted personal interviews. Participants who were alive had a main interview or no interview; in case of participants who were deceased, an End of Life interview was administered to their proxy. If participants could not be contacted, interviews were missing. The main interview was face-to-face and took place in the participant’s household, or nursing home or unknown setting. It comprised various sections (e.g., demographics, physical health and handgrip). For each section, participants were direct respondents or respondents by proxy.

In the present study, we used data from Wave 5 (baseline) and Wave 6 (follow-up), which were collected in 2013 and 2015, respectively [26, 27]. We chose Wave 5 as baseline, because it collected information on pain at the time of the interview and, in the case of joint pain, also in the previous 6 months. In the present study, we included only community-dwelling adults ≥ 50 years who had a main interview in both Wave 5 and 6. We excluded residents in the Netherlands as they participated to Wave 5 but did not participate to Wave 6.

**Fig. 1:**
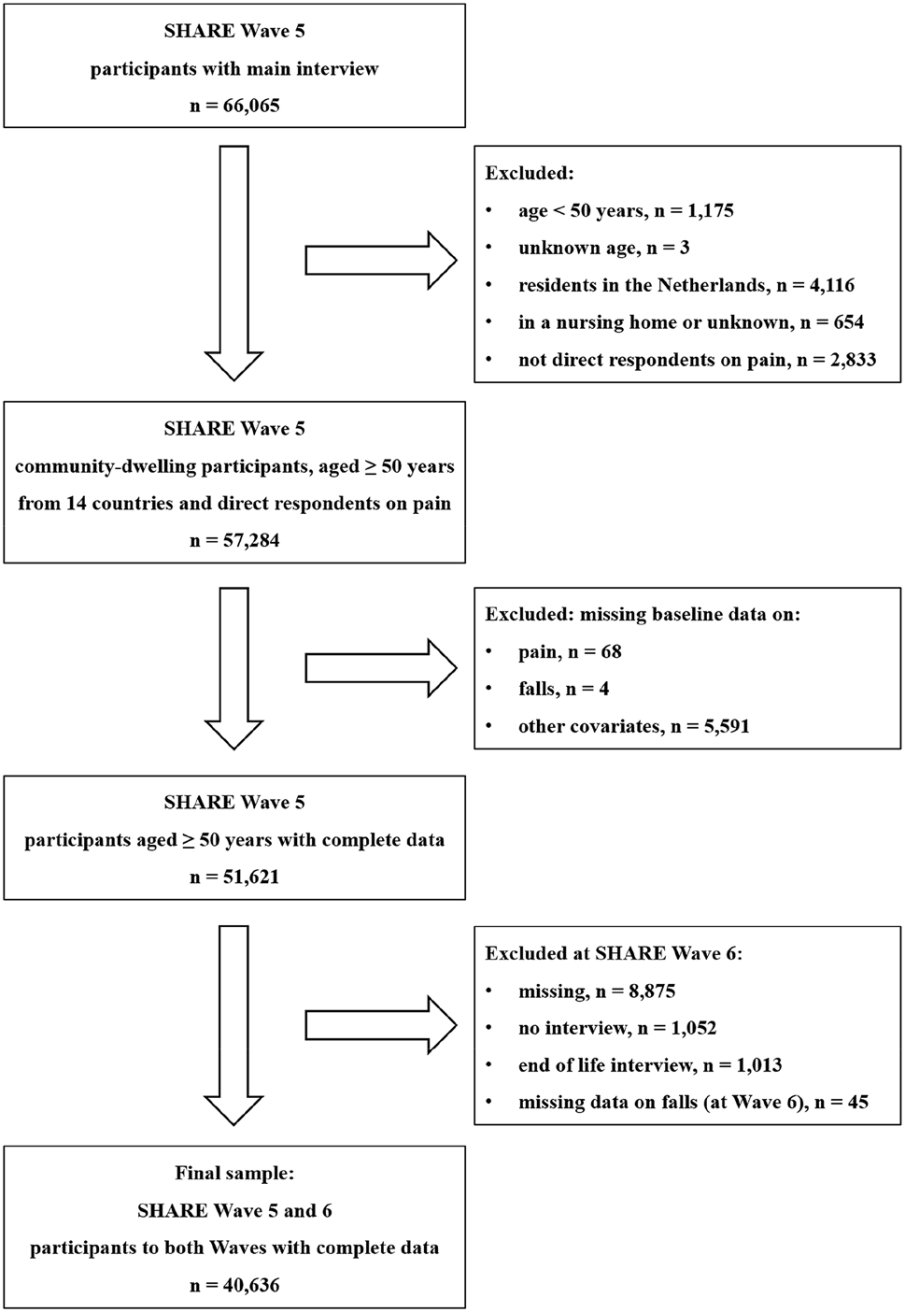
Flow-chart of study inclusion criteria. *SHARE* Survey of Health, Ageing and Retirement in Europe, *n* number. Please, note that this paper is based on data on SHARE Wave 5 and 6 Release version: 8.0.0 (10th February 2022). The numbers of participants slightly differ from those reported in a previous paper of ours that used data of Release version: 7.1.0 (26th June 2020). Every time a new SHARE wave is released, all previous waves get updated. Sometimes participants may decide to drop out of the survey and request to have all information deleted. Other observations may be deleted due to implausible or inconsistent reporting

Figure 1 shows the study flow chart. At Wave 5 (baseline), 66,065 participants had a main interview. We excluded those younger than 50 years (*n* = 1175) or of unknown age (*n* = 3), those residents in the Netherlands (*n* = 4116), those interviewed in a nursing home or unknown setting (*n* = 654), those who were not direct respondents on pain (*n* = 2833) and those with missing data on pain (*n* = 68) or falls (*n* = 4) or co-variates of interest at baseline (*n* = 5591). At follow-up (Wave 6), we further excluded 10,985 participants without a main interview or data on falls. Therefore, this study included 40,636 community-dwelling adults ≥ 50 years at baseline, who were direct respondent on pain and with complete data of interest. They were residents from 14 countries: Austria, Belgium, Czech Republic, Denmark, Estonia, France, Germany, Israel, Italy, Luxembourg, Slovenia, Spain, Sweden and Switzerland.

Those participants who were lost to follow up between Wave 5 and 6 were more likely to be men, and report moderate or severe pain and falls at baseline (Wave 5), compared to those participants who were included in our study. Those who were lost to follow up and those included in our study did not differ with regard to most other baseline characteristics (i.e., collected at Wave 5), such as age and number of pain sites (Supplementary Table 1).

### Demographic and clinical characteristics

At baseline (Wave 5), socio-demographic (age, sex and country) and clinical characteristics (self-rated health, height, weight, co-morbidities, medications, vision, hearing, living alone, physical inactivity and handgrip strength) were collected.

We dichotomised self-rated health as good (“*excellent*” or “*very good*” or “*good*”) versus poor (“*fair*” or “*poor*”). Body mass index (BMI, Kg/m2) was calculated from self-reported height and weight. BMI categories were defined as follows: underweight (BMI < 18.5 kg/m^2^), normal weight (BMI ≥ 18.5 and < 25 kg/m^2^), overweight (BMI ≥ 25 and < 30 kg/m^2^)) and obesity (BMI ≥ 30 kg/m^2^) [28].

Co-morbidities were assessed by asking the participants: *“Has a doctor ever told you that you had/do you currently have any of the conditions on this card?”*; a show-card with multiple non-mutually exclusive options was presented to the participants. Based on previous literature [9], we selected these co-morbidities: heart attack, hypertension, high cholesterol, stroke, diabetes, chronic lung disease, cancer, Parkinson’s, cataracts, hip fracture, other fractures, cognitive decline, affective/emotional disorder, rheumatoid arthritis, osteoarthritis/other rheumatism.

Medications were assessed by asking: *“Do you currently take drugs, at least once a week, for problems mentioned on this card?”* and a list was shown. Based on previous literature [9], we selected these medications: anti-hypertensives, drugs for joint pain, drugs for other pain, drugs for sleep, drugs for anxiety or depression, drugs for suppressing inflammation (only glucocorticoids or steroids).

We classified self-reported vision as good (distant and close vision were “*excellent*”, “*very good*” or “*good*”) versus poor (distant or close vision were “*fair*” or “*poor*”). Similarly, we classified self-reported hearing as good versus poor. Participants were asked how many people lived in their household and were classified as living alone versus not.

Participants were asked: “*How often do you engage in vigorous physical activity, such as sports, heavy housework, or a job that involves physical labour?*” and “*How often do you engage in activities that require a moderate level of energy such as gardening, cleaning the car, or doing a walk?*”. Physical inactivity was defined as neither vigorous nor moderate physical activity. Handgrip strength was measured with a dynamometer, as previously described [29]; we classified the participants into sex-specific tertiles of maximum grip strength.

### Pain

At baseline (Wave 5), participants were asked: “*Are you troubled with pain?*”, options were “*yes*” or “*no*”. Participants reporting pain were asked to classify the intensity of pain as “*mild*”, “*moderate*” or “*severe*”. They were then asked about the location of pain: “*In which parts of the body do you feel pain?*”; options were “*back*”, “*hips*”, “*knees*”, “*other joints*”, “*mouth/teeth*”, “*other parts of the body, but not joints*” and “*all over*”; participants could select more than one option. We computed the number of pain sites. Participants reporting joint pain were asked “*You have just told me that you are bothered by pain in your back, knees, hips or another joint. Have you been bothered for the past six months at least by any of these joint pains?*”; options were “*yes*” or “*no*”.

### Falls

At baseline (Wave 5), previous falls were assessed by asking the participants: *“For the past six months at least, have you been bothered by any of the health conditions on this card?*” options included: *“falling down”*. At follow-up (Wave 6), participants were again asked about falls in the 6 months preceding the interview. At baseline and follow-up, respectively, we dichotomised the participants as those reporting no falls versus those reporting one or more falls.

### Statistical analyses

All analyses were performed using SPSS software (version 25). We reported the baseline characteristics of our study population by: (1) sex, (2) age groups, (3) intensity of pain, (4) number of pain sites and (5) presence of back pain. We tested for differences in baseline characteristics using Pearson’s chi-square test for categorical variables and student’s *t* test or analysis of variance (ANOVA), as appropriate, for continuous variables. We considered a *p* value ≤ 0.05 to be statistically significant.

Binary logistic regression models were used to assess the cross-sectional and longitudinal associations between intensity of pain at baseline and falls risk at baseline and follow-up, respectively. The category “no pain” was set as the reference. We performed our analyses in four steps. In Model 1, we adjusted our analyses for age (continuous variable) and sex. In Model 2, we further adjusted for self-rated health, BMI category, co-morbidities, vision, hearing, living alone, country and, in longitudinal analyses, previous falls. In Model 3, we adjusted for all co-variates of Model 2 and medications. In Model 4, we further adjusted for physical inactivity and sex-specific tertiles of grip strength. We entered each and every co-morbidity and medication separately. Medications were added to Model 3 and not 2 as their prescribing may vary by country.

Further analyses were performed after stratifying the participants into three age groups: aged 50–64 years; aged 65–79 years and aged ≥ 80 years. These age groups are commonly used in the literature [15, 23, 24]. We computed interaction terms as age (as a continuous variable) by intensity of pain (as coded as follows: 1 = no pain; 2 = mild pain; 3 = moderate pain; 4 = severe pain), to test whether age may modify the associations between intensity of pain and falls risk.

Secondary analyses were performed after excluding participants who reported previous falls, or hip fractures or other fractures at baseline, to account for possible reverse causation in the association between pain and falls.

We used binary logistic regression models to explore the longitudinal associations between number of pain sites and falls risk at follow-up. Furthermore, we explored the longitudinal associations between presence of pain at each specific anatomic site (e.g., back pain versus no back pain) and falls risk at follow-up. In detail, we also explored whether participants with back pain had an increased falls risk, at follow-up, compared to those *without back pain* (rather than those *without any pain*), in line with previous reports [23, 24]. Likewise, we tested whether presence versus absence of pain of hips at baseline was associated with an increased falls risk at follow-up. Similar analyses were carried out for each specific anatomic sites.

### Ethics

SHARE has been repeatedly reviewed and approved by the Ethics Committee of the University of Mannheim, during Waves 1 to 4, and by the Ethics Council of the Max Planck Society for Wave 4 and the continuation of the project [for details, 25–27]. All participants provided a written informed consent [25–27].

## Results

### Characteristics at baseline

Baseline interviews were conducted from January to December 2013. Our study population included 40,636 community-dwelling adults; 22,486 (55.3%) were women. Age ranged from 50 to 103 years; mean age was 65.8 (standard deviation 9.3) years. Baseline characteristics differed between men and women (Supplementary Table 2) and between age groups (Supplementary Table 3). Women were more likely to report pain, higher intensity of pain, multisite pain and pain in each specific anatomic site than men (Supplementary Table 2). Participants aged ≥ 80 years were more likely to report pain, severe pain, multisite pain and pain at each anatomic site except mouth/teeth, compared to younger participants (Supplementary Table 3).

### Intensity of pain at baseline

In total, 23,932 (58.9%) participants reported no pain, 4,010 (9.9%) mild pain, 9,249 (22.8%) moderate pain and 3,445 (8.5%) severe pain (Table 1). The distribution of intensity of pain varied by sex and country. For example, severe pain was reported by 2.8% men and 5.2% women in Switzerland versus 12.8% men and 18.4% women in France (Supplementary Tables 4, 5).

**Table 1:** Characteristics of study population at baseline stratified by intensity of pain

	All (*n* = 40,636)	No pain (*n* = 23,932)	Mild pain (*n* = 4010)	Moderate pain (*n* = 9249)	Severe pain (*n* = 3445)	*P* value
Age (years), mean (SD)	65.8 (9.3)	65.2 (9.1)	65.6 (9.0)	67.1 (9.5)	67.1 (9.7)	< 0.001
Women, *n* (%)	22,486 (55.3)	12,105 (50.6)	2,260 (56.4)	5,832 (63.1)	2,289 (66.4)	< 0.001
Self-rated health, *n* (%)						
Good	26,975 (66.4)	19,225 (80.3)	2,787 (69.5)	4,145 (44.8)	818 (23.7)	< 0.001
Poor	13,661 (33.6)	4,707 (19.7)	1,223 (30.5)	5,104 (55.2)	2,627 (76.3)	
BMI category, *n* (%)						
Underweight	431 (1.1)	264 (1.1)	31 (0.8)	87 (0.9)	49 (1.4)	< 0.001
Normal	14,550 (35.8)	9,512 (39.7)	1,412 (35.2)	2,712 (29.3)	914 (26.5)	
Overweight	16,884 (41.5)	10,072 (42.1)	1,713 (42.7)	3,839 (41.5)	1,260 (36.6)	
Obese	8,771 (21.6)	4,084 (17.1)	854 (21.3)	2,611 (28.2)	1,222 (35.5)	
Co-morbidities, *n* (%):						
Heart attack	4,272 (10.5)	1,956 (8.2)	379 (9.5)	1,266 (13.7)	671 (19.5)	< 0.001
Hypertension	16,127 (39.7)	8,322 (34.8)	1,626 (40.5)	4,421 (47.8)	1,758 (51.0)	< 0.001
High cholesterol	9,609 (23.6)	4,969 (20.8)	1,001 (25.0)	2,537 (27.4)	1,102 (32.0)	< 0.001
Stroke	1,298 (3.2)	577 (2.4)	90 (2.2)	407 (4.4)	224 (6.5)	< 0.001
Diabetes	4,924 (12.1)	2,340 (9.8)	477 (11.9)	1,430 (15.5)	677 (19.7)	< 0.001
Chronic lung disease	2,285 (5.6)	933 (3.9)	185 (4.6)	758 (8.2)	409 (11.9)	< 0.001
Cancer	2,066 (5.1)	1,084 (4.5)	173 (4.3)	517 (5.6)	292 (8.5)	< 0.001
Parkinson’s disease	230 (0.6)	94 (0.4)	18 (0.4)	72 (0.8)	46 (1.3)	< 0.001
Cataracts	3,324 (8.2)	1,485 (6.2)	333 (8.3)	1,000 (10.8)	506 (14.7)	< 0.001
Hip fracture	620 (1.5)	249 (1.0)	66 (1.6)	203 (2.2)	102 (3.0)	< 0.001
Other fracture	2,409 (5.9)	1,026 (4.3)	248 (6.2)	732 (7.9)	403 (11.7)	< 0.001
Cognitive decline	214 (0.5)	82 (0.3)	21 (0.5)	74 (0.8)	37 (1.1)	< 0.001
Affective/emotional	2,185 (5.4)	798 (3.3)	172 (4.3)	766 (8.3)	449 (13.0)	< 0.001
Rheumatoid arthritis	3,463 (8.5)	704 (2.9)	405 (10.1)	1,542 (16.7)	812 (23.6)	< 0.001
Osteoarthritis/other rheumatism	7,284 (17.9)	1,934 (8.1)	891 (22.2)	3,030 (32.8)	1,429 (41.5)	< 0.001
Drugs, *n* (%)						
Anti-hypertensives	17,087 (42.0)	8,842 (36.9)	1,703 (42.5)	4,658 (50.4)	1,884 (54.7)	< 0.001
Drugs for joint pain	6,315 (15.5)	891 (3.7)	645 (16.1)	3,089 (33.4)	1,690 (49.1)	< 0.001
Drugs for other pain	4,539 (11.2)	763 (3.2)	498 (12.4)	2,040 (22.1)	1,238 (35.9)	< 0.001
Drugs for sleep	2,918 (7.2)	1,005 (4.2)	244 (6.1)	1,057 (11.4)	612 (17.8)	< 0.001
Drugs for anxiety or depression	2,412 (5.9)	956 (4.0)	196 (4.9)	799 (8.6)	461 (13.4)	< 0.001
Drugs for suppressing inflammation (only glucocorticoids or steroids)	1,175 (2.9)	255 (1.1)	120 (3.0)	511 (5.5)	289 (8.4)	< 0.001
Previous fall(s)	2,503 (6.2)	799 (3.3)	231 (5.8)	897 (9.7)	576 (16.7)	< 0.001
Physical inactivity, *n* (%)	3,039 (7.5)	1,063 (4.4)	234 (5.8)	1,044 (11.3)	698 (20.3)	< 0.001
Lives alone, *n* (%)	8,379 (20.6)	4,635 (19.4)	801 (20.0)	2,080 (22.5)	863 (25.1)	< 0.001
Poor vision, *n* (%)	9,825 (24.2)	4,864 (20.3)	981 (24.5)	2,797 (30.2)	1,183 (34.3)	< 0.001
Poor hearing, *n* (%)	7,434 (18.3)	3,616 (15.1)	754 (18.8)	2,153 (23.3)	911 (26.4)	< 0.001
Maximum grip strength (Kg/m2), mean (SD)	34.0 (11.7)	35.8 (11.4)	33.9 (11.6)	31.1 (11.5)	29.3 (11.8)	< 0.001
Grip strength tertile^a^, *n* (%)						
Low	12,988 (32.0)	6,469 (27.0)	1,248 (31.1)	3,662 (39.6)	1,609 (46.7)	< 0.001
Middle	13,958 (34.3)	8,377 (35.0)	1,436 (35.8)	3,094 (33.5)	1,051 (30.5)	
High	13,690 (33.7)	9,086 (38.0)	1,326 (33.1)	2,493 (27.0)	785 (22.8)	

*n* number, *SD* standard deviation. *P* values were computed by Pearson’s chi-square for categorical variables and by ANOVA for continuous variables. Visual inspection showed that age and maximum grip strength were roughly normally distributed in the study population

^a^Sex-specific

Participants with severe pain were older, more likely to be women and to report poor health, obesity, co-morbidities, use of medications, physical inactivity, living alone, poor vision, poor hearing and to have poorer handgrip strength, compared to those without pain (Table 1). The proportion of women, of participants living alone, and of participants reporting poor health, obesity, co-morbidities, use of medications, physical inactivity, poor vision and poor hearing were higher with increasing intensity of pain, being lowest in those with no pain, intermediate in those with mild and moderate pain and highest in those with severe pain (Table 1). The proportion of participants in the lowest sex-specific handgrip strength tertile increased with increasing intensity of pain (Table 1).

Participants with multisite pain were older, more likely to be women, to report poor health, obesity, co-morbidities, use of medications, physical inactivity, living alone, sensory impairments and to have poorer handgrip strength, compared to those with no pain or pain in one site only (Table 2).

**Table 2:** Characteristics of study population at baseline by number of pain sites

	No pain (*n* = 23,932)	1 site (*n* = 8425)	≥ 2 sites or all over (*n* = 8279)	*P* value
Age (years), mean (SD)	65.2 (9.1)	66.2 (9.4)	67.3 (9.5)	< 0.001
Women, *n* (%)	12,105 (50.6)	4,812 (57.1)	5,569 (67.3)	< 0.001
Self-rated health, *n* (%)				
Good	19,225 (80.3)	4,830 (57.3)	2,920 (35.3)	< 0.001
Poor	4,707 (19.7)	3,595 (42.7)	5,359 (64.7)	
BMI category, *n* (%)				
Underweight	264 (1.1)	90 (1.1)	77 (0.9)	< 0.001
Normal	9,512 (39.7)	2,754 (32.7)	2,284 (27.6)	
Overweight	10,072 (42.1)	3,465 (41.1)	3,347 (40.4)	
Obese	4,084 (17.1)	2,116 (25.1)	2,571 (31.1)	
Co-morbidities, *n* (%)				
Heart attack	1,956 (8.2)	947 (11.2)	1,369 (16.5)	< 0.001
Hypertension	8,322 (34.8)	3,575 (42.4)	4,230 (51.1)	< 0.001
High cholesterol	4,969 (20.8)	2,047 (24.3)	2,593 (31.3)	< 0.001
Stroke	577 (2.4)	278 (3.3)	443 (5.4)	< 0.001
Diabetes	2,340 (9.8)	1,164 (13.8)	1,420 (17.2)	< 0.001
Chronic lung disease	933 (3.9)	538 (6.4)	814 (9.8)	< 0.001
Cancer	1,084 (4.5)	471 (5.6)	511 (6.2)	< 0.001
Parkinson’s disease	94 (0.4)	55 (0.7)	81 (1.0)	< 0.001
Cataracts	1,485 (6.2)	705 (8.4)	1,134 (13.7)	< 0.001
Hip fracture	249 (1.0)	144 (1.7)	227 (2.7)	< 0.001
Other fracture	1,026 (4.3)	551 (6.5)	832 (10.0)	< 0.001
Cognitive decline	82 (0.3)	45 (0.5)	87 (1.1)	< 0.001
Affective/emotional	798 (3.3)	464 (5.5)	923 (11.1)	< 0.001
Rheumatoid arthritis	704 (2.9)	811 (9.6)	1,948 (23.5)	< 0.001
Osteoarthritis/other rheumatism	1,934 (8.1)	1,917 (22.8)	3,433 (41.5)	< 0.001
Drugs, *n* (%)				
Anti-hypertensives	8,842 (36.9)	3,839 (45.6)	4,406 (53.2)	< 0.001
Drugs for joint pain	891 (3.7)	1,704 (20.2)	3,720 (44.9)	< 0.001
Drugs for other pain	763 (3.2)	1,321 (15.7)	2,455 (29.7)	< 0.001
Drugs for sleep	1,005 (4.2)	648 (7.7)	1,265 (15.3)	< 0.001
Drugs for anxiety or depression	956 (4.0)	511 (6.1)	945 (11.4)	< 0.001
Drugs for suppressing inflammation (only glucocorticoids or steroids)	255 (1.1)	320 (3.8)	600 (7.2)	< 0.001
Previous fall(s)	799 (3.3)	590 (7.0)	1,114 (13.5)	< 0.001
Physical inactivity, *n* (%)	1,063 (4.4)	794 (9.4)	1,182 (14.3)	< 0.001
Lives alone, *n* (%)	4,635 (19.4)	1,751 (20.8)	1,993 (24.1)	< 0.001
Poor vision, *n* (%)	4,864 (20.3)	2,180 (25.9)	2,781 (33.6)	< 0.001
Poor hearing, *n* (%)	3,616 (15.1)	1,729 (20.5)	2,089 (25.2)	< 0.001
Maximum handgrip strength (Kg/m2), mean (SD)	35.8 (11.4)	33.3 (11.6)	29.5 (11.5)	< 0.001
Handgrip strength tertile^a^, *n* (%)				
Low	6,469 (27.0)	2,815 (33.4)	3,704 (44.7)	< 0.001
Middle	8,377 (35.0)	3,023 (35.9)	2,558 (30.9)	
High	9,086 (38.0)	2,587 (30.7)	2,017 (24.4)	

*n* number, *SD* standard deviation. *P* values were computed by Pearson’s chi-square for categorical variables and by ANOVA for continuous variables

^a^Sex-specific

The proportion of participants reporting pain at each anatomic site (i.e., back; hips; knees; other joints; mouth/teeth; other, not joints; all over) increased alongside intensity of pain (Supplementary Table 6). Back pain was the most frequently reported site of pain in all groups, with a frequency of 45.5% in participants with mild pain, 53.2% in those with moderate pain and 57.5% in those with severe pain (Supplementary Table 6). The proportion of participants with multisite pain was highest in those with severe pain (*n* = 2,151, 62.4%, Supplementary Table 6). Of the 16,704 participants reporting pain, 13,149 (78.7%) participants reported joint pain at the time of baseline interview and in the previous 6 months (Supplementary Table 6).

### Falls at baseline

At baseline, 2503 (6.2%) participants—777 (4.3%) men and 1726 (7.7%) women—reported falls in the previous 6 months. The frequency of history of falls varied by country in both sexes (Supplementary Table 7) and increased with increasing age (Supplementary Table 3).

In cross-sectional analyses, participants with mild, moderate and severe baseline pain were more likely to report falling in the preceding 6 months, with likelihood [OR (95% CI)] of 1.36 (1.16–1.59), 1.59 (1.41–1.78) and 2.11 (1.83–2.44), respectively, compared to those without pain, after full adjustment (Supplementary Table 8). These findings were consistent in both sexes.

### Falls at follow-up

Follow-up interviews were conducted from January to November 2015. Mean time interval between baseline and follow-up interview was 23.6 (SD 3.4) months. At follow-up, 2805 (6.9%) participants—907 (5.0%) men and 1898 (8.4%) women—reported fall(s) in the previous 6 months. In detail, 764 (3.9%) participants aged 50–64 years, 1426 (8.2%) participants aged 65–79 years and 615 (16.9%) participants aged ≥ 80 years reported falls.

### Intensity of baseline pain and falls risk at follow-up

In age- and sex-adjusted analyses (Model 1), participants with mild, moderate and severe baseline pain had an increased falls risk of 1.32 (1.14–1.52), 2.04 (1.86–2.24) and 3.22 (2.87–3.61), respectively, compared to those without pain (Table 3).

**Table 3:** Longitudinal association between intensity of pain and falls risk at follow-up

	All (*n* = 40,636)			Men (*n* = 18,150)			Women (*n* = 22,486)		
	*n* of falls	OR [95% CI]	*P* value	n of falls	OR [95% CI]	*P* value	*n* of falls	OR [95% CI]	*P* value
Model 1									
No pain	1,083	1 (ref)		415	1 (ref)		668	1 (ref)	
Mild pain	247	1.32 [1.14; 1.52]	< 0.001	82	1.34 [1.05; 1.72]	0.018	165	1.30 [1.09; 1.55]	0.004
Moderate pain	949	2.04 [1.86; 2.24]	< 0.001	277	2.23 [1.90; 2.61]	< 0.001	672	1.95 [1.74; 2.19]	< 0.001
Severe pain	526	3.22 [2.87; 3.61]	< 0.001	133	3.42 [2.77; 4.21]	< 0.001	393	3.12 [2.72; 3.58]	< 0.001
Model 2									
No pain	1,083	1 (ref)		415	1 (ref)		668	1 (ref)	
Mild pain	247	1.12 [0.97; 1.30]	0.132	82	1.10 [0.85; 1.42]	0.486	165	1.12 [0.93; 1.35]	0.231
Moderate pain	949	1.43 [1.29; 1.59]	< 0.001	277	1.49 [1.24; 1.79]	< 0.001	672	1.39 [1.22; 1.58]	< 0.001
Severe pain	526	1.70 [1.48; 1.94]	< 0.001	133	1.68 [1.31; 2.15]	< 0.001	393	1.71 [1.46; 2.02]	< 0.001
Model 3									
No pain	1,083	1 (ref)		415	1 (ref)		668	1 (ref)	
Mild pain	247	1.11 [0.95; 1.28]	0.191	82	1.07 [0.83; 1.39]	0.584	165	1.10 [0.92; 1.33]	0.299
Moderate pain	949	1.37 [1.23; 1.53]	< 0.001	277	1.43 [1.19; 1.73]	< 0.001	672	1.33 [1.16; 1.52]	< 0.001
Severe pain	526	1.58 [1.37; 1.82]	< 0.001	133	1.57 [1.21; 2.05]	0.001	393	1.58 [1.33; 1.88]	< 0.001
Model 4									
No pain	1,083	1 (ref)		415	1 (ref)		668	1 (ref)	
Mild pain	247	1.10 [0.95; 1.28]	0.197	82	1.08 [0.83; 1.40]	0.558	165	1.10 [0.91; 1.32]	0.330
Moderate pain	949	1.35 [1.21; 1.51]	< 0.001	277	1.42 [1.18; 1.72]	< 0.001	672	1.30 [1.14; 1.49]	< 0.001
Severe pain	526	1.52 [1.31; 1.75]	< 0.001	133	1.53 [1.18; 2.00]	0.001	393	1.51 [1.27; 1.80]	< 0.001

Odds ratios and 95% confidence intervals were calculated by binary logistic regression. Model 1: adjusted for age and sex. Model 2: Model 1 + self-rated health, BMI category, heart attack, hypertension, high cholesterol, stroke, diabetes, chronic lung disease, cancer, Parkinson’s, cataracts, hip fracture, other fractures, cognitive impairment, affective/emotional disorder, rheumatoid arthritis, osteoarthritis or other rheumatism, poor vision, poor hearing, lives alone, previous falls, country. Model 3: Model 2 + anti-hypertensives; drugs for joint pain; drugs for other pain; drugs for sleep; drugs for anxiety or depression; drugs for suppressing inflammation (only glucocorticoids or steroids). Model 4: Model 3 + physical inactivity, sex-specific tertiles of handgrip strength. Number of participants reporting fall(s) at follow-up in each category: all participants: 2,805; men: 907; women: 1,898

*OR* odds ratios, *CI* confidence intervals, *ref* reference

After further adjusting for self-rated health, BMI category, co-morbidities, vision, hearing, living alone, country and previous falls (Model 2), moderate and severe pain remained associated with an increased falls risk of 1.43 (1.29–1.59) and 1.70 (1.48–1.94), respectively, compared to no pain (Table 3). These associations remained significant after further adjusting for medications (Model 3, Table 3).

After full adjustment (Model 4), participants with moderate pain and those with severe pain had an increased falls risk of 1.35 (1.21–1.51) and 1.52 (1.31–1.75), respectively, compared to those without pain (Table 3). Findings were consistent in both sexes, in all Models of adjustment.

The longitudinal association between intensity of pain and falls risk varied by age (all p for interaction < 0.001, Supplementary Table 9). After full adjustment, moderate pain was prospectively associated with an increased falls risk of 1.64 (1.34–2.02) and 1.36 (1.17–1.59) in participants aged 50–64 years and those aged 65–79 years, respectively, while no significant association was found in those aged ≥ 80 years (OR 0.99, 95% CI 0.77–1.26). After full adjustment, severe pain was longitudinally associated with an increased falls risk of 1.76 (1.34–2.30) and 1.56 (1.27–1.92) in participants aged 50–64 years and those aged 65–79 years, respectively, while no significant association was found in those aged ≥ 80 years (OR 1.09, 95% CI 0.79–1.51).

The associations between moderate and severe baseline pain and increased falls risk at follow-up remained significant in participants without history of falls, hip or other fracture at baseline (Supplementary Table 10).

### Number of sites of pain and falls risk at follow-up

After full adjustment (Model 4), participants with pain in one site and those with multisite pain had an increased falls risk of 1.19 (1.06–1.33) and 1.48 (1.31–1.66), respectively, compared to those without pain (Table 4). Among participants with pain at baseline, participants with multisite pain had an increased falls risk of 1.29 (1.14–1.45) compared to those with pain in one site (Supplementary Table 11). These findings were consistent in both sexes.

**Table 4:** Longitudinal association between number of pain sites and falls risk at follow-up

	All (*n* = 40,636)			Men (*n* = 18,150)			Women (*n* = 22,486)		
	*n* of falls	OR [95% CI]	*P* value	*n* of falls	OR [95% CI]	*P* value	*n* of falls	OR [95% CI]	*P* value
Model 1									
No pain	1,083	1 (ref)		415	1 (ref)		668	1 (ref)	
1 site	628	1.54 [1.39; 1.71]	< 0.001	195	1.52 [1.27; 1.81]	< 0.001	433	1.55 [1.36; 1.76]	< 0.001
≥ 2 sites or all over	1,094	2.68 [2.44; 2.93]	< 0.001	297	3.11 [2.66; 3.64]	< 0.001	797	2.50 [2.24; 2.80]	< 0.001
Model 2									
No pain	1,083	1 (ref)		415	1 (ref)		668	1 (ref)	
1 site	628	1.23 [1.10; 1.37]	< 0.001	195	1.18 [0.98; 1.42]	0.088	433	1.24 [1.08; 1.42]	0.002
≥ 2 sites or all over	1,094	1.61 [1.44; 1.79]	< 0.001	297	1.76 [1.45; 2.13]	< 0.001	797	1.54 [1.34; 1.76]	< 0.001
Model 3									
No pain	1,083	1 (ref)		415	1 (ref)		668	1 (ref)	
1 site	628	1.20 [1.07; 1.34]	0.001	195	1.15 [0.95; 1.40]	0.143	433	1.21 [1.05; 1.38]	0.007
≥ 2 sites or all over	1,094	1.51 [1.35; 1.70]	< 0.001	297	1.68 [1.36; 2.06]	< 0.001	797	1.43 [1.24; 1.65]	< 0.001
Model 4									
No pain	1,083	1 (ref)		415	1 (ref)		668	1 (ref)	
1 site	628	1.19 [1.06; 1.33]	0.002	195	1.15 [0.95; 1.40]	0.151	433	1.19 [1.04; 1.37]	0.013
≥ 2 sites or all over	1,094	1.48 [1.31; 1.66]	< 0.001	297	1.65 [1.34; 2.03]	< 0.001	797	1.39 [1.21; 1.61]	< 0.001

Odds ratios and 95% confidence intervals were calculated by binary logistic regression. Model 1: adjusted for age and sex. Model 2: Model 1 + self-rated health, BMI category, heart attack, hypertension, high cholesterol, stroke, diabetes, chronic lung disease, cancer, Parkinson’s, cataracts, hip fracture, other fractures, cognitive impairment, affective/emotional disorder, rheumatoid arthritis, osteoarthritis or other rheumatism, poor vision, poor hearing, lives alone, previous falls, country. Model 3: Model 2 + anti-hypertensives; drugs for joint pain; drugs for other pain; drugs for sleep; drugs for anxiety or depression; drugs for suppressing inflammation (only glucocorticoids or steroids). Model 4: Model 3 + physical inactivity, sex-specific tertiles of handgrip strength. Number of participants reporting fall(s) at follow-up in each category: all participants: 2,805; men: 907; women: 1,898

*OR* odds ratios, *CI* confidence intervals, *ref* reference

### Site of pain and falls risk at follow-up

**Fig. 2:**
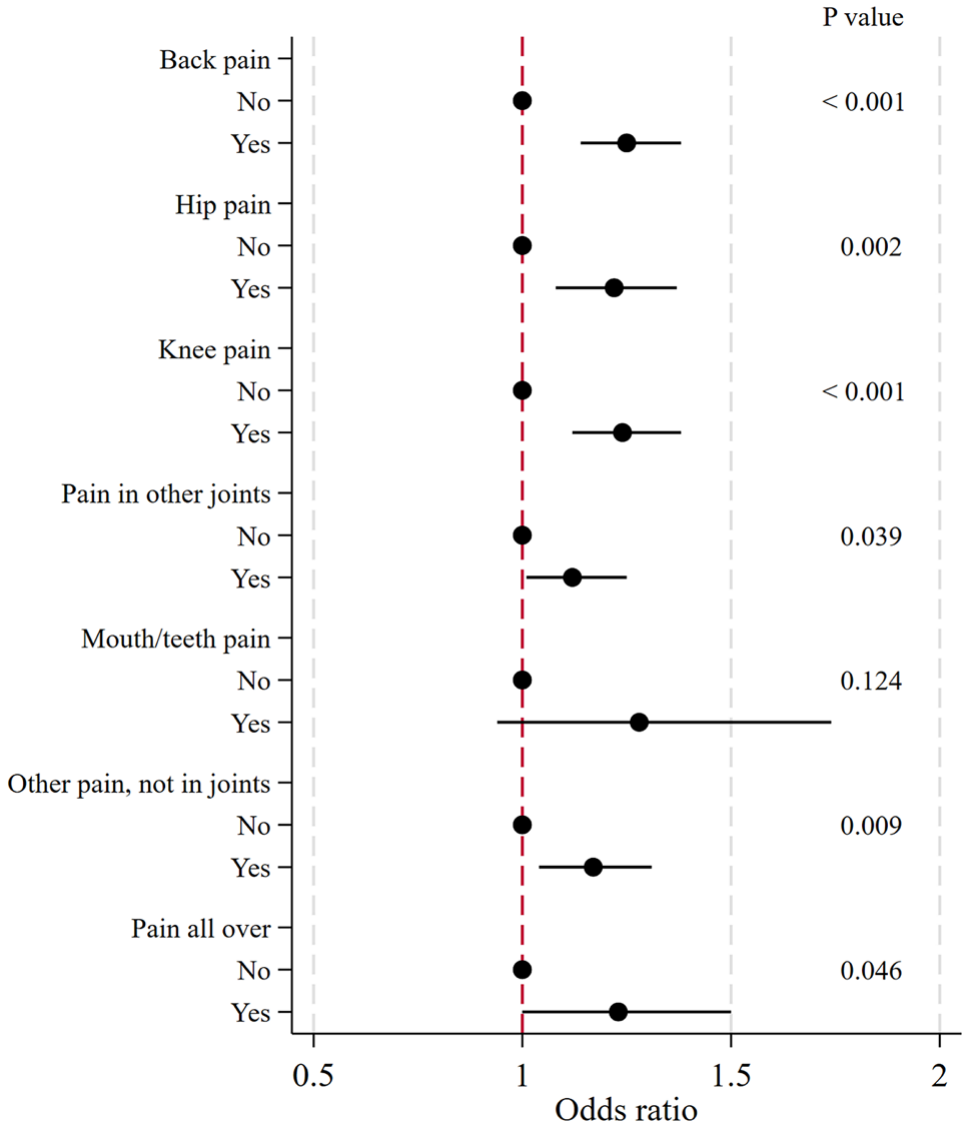
Longitudinal association between presence of pain at specific anatomic sites and falls risk at follow-up. Dots and bars represent odds ratios and 95% confidence intervals, respectively. Odds ratios and 95% confidence intervals were calculated by binary logistic regression. All analyses were adjusted for Model 4: adjusted for age, sex, self-rated health, BMI category, heart attack, hypertension, high cholesterol, stroke, diabetes, chronic lung disease, cancer, Parkinson’s, cataracts, hip fracture, other fractures, cognitive impairment, affective/emotional disorder, rheumatoid arthritis, osteoarthritis or other rheumatism, poor vision, poor hearing, lives alone, previous falls, country, anti-hypertensives, drugs for joint pain, drugs for other pain, drugs for sleep, drugs for anxiety or depression, drugs for suppressing inflammation (only glucocorticoids or steroids), physical inactivity, sex-specific tertiles of grip strength. Number of participants reporting fall(s) at follow-up: 2,805

After full adjustment, presence of back pain was associated with an increased falls risk at follow-up, compared to absence of back pain (Fig. 2). Similar associations were observed for presence versus absence of pain at each anatomic site, but not significant for pain of mouth/teeth (Fig. 2).

## Discussion

In this large, longitudinal, cross-national study, greater intensity of pain at baseline was associated with an increased risk of subsequent falls in community-dwelling adults ≥ 50 years, at 2-year follow-up. Moderate and severe pain were associated with an increased falls risk, compared to no pain, independently of socio-demographic and clinical variables. These associations were more pronounced and consistent in adults aged < 80 years. Furthermore, multiple site pain and pain at most specific anatomic sites of interest were each associated with an increased falls risk.

The novelty of our study was to show the associations between various characteristics of pain at the time of the baseline interview—intensity, number of sites, presence at specific sites—and falls risk in a large, European population. We showed a dose–response relationship between higher intensity and number of sites of pain, respectively, and increased falls risk in 6 months prior to the interview at 2-year follow-up. Finally, a major novelty was that the strength of the association between higher intensity of pain and increased falls risk varied by age, being more pronounced in the younger participants. This could have clinical implications; pain assessment and management may be more relevant for falls risk stratification and prevention in middle aged than older adults.

In line with our findings, age modified the association between back pain and risk of recurrent falls in older community-dwelling U.S. women in the Study of Osteoporotic Fractures; back pain was strongly associated with risk of recurrent falls among women 65–74 years but not those ≥ 75 years [23]. In contrast, age did not affect the association between back pain and falls risk in older community-dwelling U.S. men from the Osteoporotic Fractures in Men Study [24].

The prevalence of pain at the time of the interview in our study (34.8% in men and 46.2% in women) cannot be directly compared to those of chronic pain in previous studies [15, 16]. However, most of our participants with joint pain at the time of interview also had pain in the previous 6 months, suggesting an overlap between present and recurrent or chronic pain. In line with previous literature, women were more likely to report pain than men [14–17]. In our study, the prevalence of pain and moderate to severe pain increased with increasing age, while findings from previous studies were mixed [14–16].

In our study, the prevalence of falls (6–7% in 6 months prior to the baseline and follow-up interview) was lower compared to previous studies, possibly reflecting healthy respondent bias, or cohort effect (contemporary adults may be healthier than adults of the eighties [3]), or underestimation due to recall bias.

Pain may be linked to falls risk through various mechanisms. First, pain may be a symptom of underlying musculoskeletal diseases—rheumatoid arthritis, osteoarthritis and previous fractures—that directly affect mobility and falls risk [30]. Pain has been associated with loneliness, depression and anxiety, which are falls risk factors [31]. It may result from complications of diabetes as in diabetic neuropathy or of stroke in post-stroke pain; diabetes and stroke contribute to falls risk. Chronic pain has been associated with many physical and mental co-morbidities [32]. Pain has been associated with use of analgesic and psychotropic medications [33], also falls risk factors [9]. Pain of hips and knees may be related to increased weight-bearing in obesity, a falls risk factor [10, 11]. Pain and vision and hearing impairments may share common risk factors, such as diabetes. Indeed, in our study, higher intensity of pain and number of pain sites, respectively, were associated with an increasing frequency of most co-morbidities, use of medications, living alone, obesity and sensory impairments. Our associations between pain and falls risk became attenuated but remained consistent after adjusting for co-morbidities and medications (Model 2 and 3), suggesting that the associations were partly confounded but existed beyond these co-variates.

Pain may be the consequence of previous falls and fall-related fractures (reverse causation); history of falls is a major risk factor for further falls. However, our findings remained consistent after excluding participants with history of falls or fractures at baseline.

Pain may cause falls, through various pathways. Pain may induce fear of falling, activity avoidance and physical inactivity [20, 21, 34]. Indeed, older adults with chronic musculoskeletal pain are less active than those without pain [20]. Physical inactivity—through deconditioning and reduced muscle strength—may mediate the association between pain and falls [35]. Furthermore, pain is a stressor that activates the (subclinical) inflammatory response through the hypothalamic–pituitary axis. Chronic inflammation, even subclinical, has detrimental effects on muscle strength [36, 37]. Indeed, pain was associated with lower handgrip strength, as objectively measured, in our population. Handgrip strength is an inexpensive, easy-to-measure proxy for overall muscle strength; handgrip strength has a moderately high correlation with strength of the lower extremity muscles [38]. Poorer handgrip strength has been associated with slower gait speed, greater physical and functional decline and increased falls risk in adults [38–40]. Yet, in our study, the associations between pain and falls minimally changed after adjusting for physical inactivity and grip strength as possible mediators. Therefore, pain may cause falls through other causal pathways.

Pain may affect postural stability. Adults with back pain have aberrant postural responses to loss of balance, including trunk muscle stiffening, inadequate use of hip joints and increasing reliance on ankle joints to regain balance [41, 42]. Postural instability is a major determinant of falls [43]. Furthermore, pain draws on attentional resources [22], thus affecting mobility tasks requiring attention. Chronic pain has been associated with poorer cognitive function in the domains of memory, attention, mental flexibility, psychomotor speed and processing speed [44]. Focusing on pain may reduce attention and responses to trip hazards and other factors leading to falls.

Our study was consistent with the suggestion that pain itself is a falls risk factor. Of note, pain has been linked to frailty and disability in older adults in population-based studies. In the English Longitudinal Study of Ageing, men and women with mild, moderate, or severe pain were more likely to develop worsening frailty, compared to those without pain, in a dose–response way [45]. In the Canadian Study of Health and Aging, older adults with moderate or higher pain had a higher likelihood of being pre-frail and frail, versus not frail, compared to those with mild or no pain [46]. In the Concord Health and Ageing in Men Project, chronic pain was longitudinally associated with an increased risk of developing frailty in older men [47]. In an Italian study, older adults with osteoarthritis and pain were more likely to develop frailty at 4-year follow-up, compared to those with osteoarthritis but without pain [48]. Further studies showed longitudinal associations between higher number of pain sites and increased risk of onset of mobility difficulty [49, 50].

A major strength of our study was the longitudinal design with the assessment of pain preceding the ascertainment of falls at follow-up. Further strengths were the large nationally representative sample sizes, the inclusion of both men and women of a wide age range, the standardised methods for data collection, the detailed characterisation of pain and the adjustment for many covariates using four models of adjustment. We stratified our analyses by sex, in view of the vast literature on sex-differences in falls and pain [7, 17]. We explored the association between pain and falls in middle-aged adults, while previous studies recruited only older adults [15, 23, 24]. Of note, we showed that age modulated the association between intensity of pain and falls risk. Although ethnicity was not reported, our study included a diverse population across European countries and Israel. We showed a significant cross-country variation in self-reported falls and pain intensity, possibly reflecting the interplay of cultural factors, varying prevalence of co-morbidities and different social and healthcare systems between countries. Using four models of adjustment, we showed that the associations between pain and falls were partly confounded but not fully explained by major falls risk factors (physical and mental co-morbidities and others, Model 2; medications, Model 3) and potential casual mediators (physical inactivity and handgrip strength, Model 4). Furthermore, the retention rate between Wave 5 and 6 was high (40,636/51,621 participants, 78.7%); the participants excluded at follow-up and those included in the final sample did not differ with regard to most baseline characteristics (i.e., characteristics that were collected at Wave 5). Therefore, we deemed attrition bias minimal and unlikely to affect our findings.

Finally, we assessed various characteristics of pain—intensity, number of sites of pain, pain in specific anatomic sites—rather than presence of pain only, using simple questions that could be used in clinical practice or other large-scale studies.

This study also had some limitations. First, our observational design did not allow us to prove causality in the association between pain and falls. We could not state that pain caused falls or that preventing, alleviating or curing pain would reduce falls risk. However, showing a longitudinal association between pain and falls—independent of co-morbidities, medications and major falls risk factor—in an observational study is the first essential step toward promoting interventional studies to assess whether better pain management may prevent falls. Further studies may explore whether different types of pain are more likely to be associated with falls risk and more amenable to treatment aimed at preventing falls. In SHARE, data on aetiology, duration and potential reversibility of pain were not available. Although pain duration was not recorded, most participants with joint pain at the time of the baseline interview also reported pain in the previous 6 months, suggesting that their pain was at least recurrent, if not chronic. A further limitation is that falls were retrospectively self-reported, possibly leading to underreporting of falls, especially non-injurious falls (recall bias). Moreover, we collected information only on falls in 6 months prior to the interview at 2-year follow-up; in this way, we may have missed falls that occurred in the months immediately following the first interview and failed to capture the association between pain and falls in the very short-term follow-up. This may have led to an underestimation of our associations. It was not recorded whether falls were recurrent or injurious. Despite adjustment for many co-variates, residual confounding could not be excluded. Finally, our study included only community-dwelling adults and its findings may not be generalizable to institutionalised populations. Replication in other populations is required.

In conclusion, pain was associated with subsequent increased falls risk in community-dwelling adults. Greater intensity of pain was associated with an increased falls risk in a dose–response way; this association was more pronounced in middle-aged adults. Multisite pain was associated with an increased falls risk compared to pain in one site only. Future qualitative studies should explore how cultural factors may influence the experience and reporting of pain in different countries. Future observational studies should explore whether pain management may differ between countries and whether this could modulate the associations between pain and falls. Future interventional studies should explore whether pain management may prevent falls.

## Supplementary Information

Below is the link to the electronic supplementary material.

Supplementary file1 (DOCX 81 KB)

## Data Availability

Data are available upon request from the SHARE website (see http://www.share-project.org/data-access/user-registration.html).
