# Supplementary material for: Association of pain and risk of falls in community-dwelling adults: a prospective study in the Survey of Health, Ageing and Retirement in Europe (SHARE)
Source: Eur Geriatr Med. 2022 Oct 13;13(6):1441–54. doi: 10.1007/s41999-022-00699-1 (PMC9722814; doi:10.1007/s41999-022-00699-1)
Supplement: Supplementary file 1 — Supplementary file1 (DOCX 81 KB) [file 41999_2022_699_MOESM1_ESM.docx]

**Article Title: Association of pain and risk of falls in community-dwelling adults: a prospective study in the Survey of Health, Ageing and Retirement in Europe (SHARE)**

**Journal Title: European Geriatric Medicine**

Giulia Ogliari^1,2^, Jesper Ryg^3,4^, Karen Andersen-Ranberg^3,4,5^, Lasse Lybecker Scheel-Hincke^5^, Jemima T. Collins^2,6,7^, Alison Cowley^6,8^, Claudio Di Lorito^6^, Louise Howe^6^, Katie R. Robinson^1,6^, Vicky Booth^6,8^, David A. Walsh^2,9,10^, John R.F. Gladman^1,2,6,11^, Rowan H. Harwood^1,2,6,11^, Tahir Masud^1,2,11^

1. Department of Health Care for Older People (HCOP), Queen’s Medical Centre, Nottingham University Hospitals NHS Trust, Nottingham, UK
2. NIHR Nottingham Biomedical Research Centre, Nottingham, UK
3. Department of Geriatric Medicine, Odense University Hospital, Odense, Denmark
4. Geriatric Research Unit, Department of Clinical Research, University of Southern Denmark, Odense, Denmark
5. Unit for Epidemiology, Biostatistics and Biodemography, Department of Public Health, University of Southern Denmark, 5000 Odense, Denmark
6. University of Nottingham, Nottingham, UK
7. University Hospitals of Derby and Burton NHS Foundation Trust, UK
8. Research & Innovation, Nottingham University Hospitals NHS Trust, Nottingham, UK
9. Pain Centre Versus Arthritis, University of Nottingham, Nottingham, UK
10. Sherwood Forest Hospitals NHS Foundation Trust, Sutton-in-Ashfield, UK
11. NIHR Applied Research Collaboration – East Midlands, Nottingham, UK

**Corresponding Author:** Giulia Ogliari, Fellow, Department of Health Care for Older People (HCOP), Queen’s Medical Centre, Nottingham University Hospitals NHS Trust, Derby Road, Nottingham, Nottinghamshire, NG7 2UH, UK, phone: +44 (0)115 924 9924 (extension 82323), e-mail: [giulia.ogliari@virgilio.it](mailto:giulia.ogliari@virgilio.it) and [Giulia.Ogliari1@nottingham.ac.uk](mailto:Giulia.Ogliari1@nottingham.ac.uk)

**Supplementary Table 1 Characteristics at baseline (Wave 5) of participants who were included in the final sample and those who were excluded between Wave 5 and 6**

|  | **Excluded at follow-up**  **(n = 10,985)** | **Final sample**  **(n = 40,636)** | **P value** |
| --- | --- | --- | --- |
| Age (years), mean (SD) | 65.7 (10.1) | 65.8 (9.3) | 0.316 |
| Women, n (%) | 5,649 (51.4) | 22,486 (55.3) | < 0.001 |
| Intensity of pain, n (%):  No pain  Mild pain  Moderate pain  Severe pain | 6,421 (58.5)  973 (8.9)  2,541 (23.1)  1,050 (9.6) | 23,932 (58.9)  4,010 (9.9)  9,249 (22.8)  3,445 (8.5) | < 0.001 |
| N of pain sites, n (%):  No pain  1 site  ≥ 2 sites or all over | 6,421 (58.5)  2,279 (20.7)  2,285 (20.8) | 23,932 (58.9)  8,425 (20.7)  8,279 (20.4) | 0.589 |
| Pain at specific site*, n (%):  Back  Hips  Knees  Other joints  Mouth / teeth  Other, not joint  All over | 2,389 (21.7)  935 (8.5)  1,630 (14.8)  1,421 (12.9)  88 (0.8)  1,178 (10.7)  293 (2.7) | 8,727 (21.5)  3,426 (8.4)  6,055 (14.9)  5,396 (13.3)  357 (0.9)  4,171 (10.3)  901 (2.2) | 0.539  0.787  0.871  0.346  0.436  0.161  0.005 |
| Self-rated health, n (%):  Good  Poor | 6,940 (63.2)  4,045 (36.8) | 26,975 (66.4)  13,661 (33.6) | < 0.001 |
| BMI category, n (%):  Underweight  Normal  Overweight  Obese | 153 (1.4)  4,207 (38.3)  4,446 (40.5)  2,179 (19.8) | 431 (1.1)  14,550 (35.8)  16,884 (41.5)  8,771 (21.6) | < 0.001 |
| Co-morbidities, n (%): |  |  |  |
| Heart attack | 1,229 (11.2) | 4,272 (10.5) | 0.042 |
| Hypertension | 4,209 (38.3) | 16,127 (39.7) | 0.009 |
| High cholesterol | 2,586 (23.5) | 9,609 (23.6) | 0.818 |
| Stroke | 381 (3.5) | 1,298 (3.2) | 0.151 |
| Diabetes | 1,337 (12.2) | 4,924 (12.1) | 0.878 |
| Chronic lung disease | 676 (6.2) | 2,285 (5.6) | 0.034 |
| Cancer | 731 (6.7) | 2,066 (5.1) | < 0.001 |
| Parkinson’s disease | 79 (0.7) | 230 (0.6) | 0.065 |
| Cataracts | 947 (8.6) | 3,324 (8.2) | 0.137 |
| Hip fracture | 203 (1.8) | 620 (1.5) | 0.017 |
| Other fracture | 662 (6.0) | 2,409 (5.9) | 0.700 |
| Cognitive decline | 101 (0.9) | 214 (0.5) | < 0.001 |
| Affective / emotional | 570 (5.2) | 2,185 (5.4) | 0.436 |
| Rheumatoid arthritis | 896 (8.2) | 3,463 (8.5) | 0.222 |
| Osteoarthritis / other rheumatism | 1,997 (18.2) | 7,284 (17.9) | 0.538 |
| Drugs, n (%) |  |  |  |
| Anti-hypertensives | 4,433 (40.4) | 17,087 (42.0) | 0.001 |
| Drugs for joint pain | 1,833 (16.7) | 6,315 (15.5) | 0.003 |
| Drugs for other pain | 1,381 (12.6) | 4,539 (11.2) | < 0.001 |
| Drugs for sleep | 852 (7.8) | 2,918 (7.2) | 0.040 |
| Drugs for anxiety or depression | 672 (6.1) | 2,412 (5.9) | 0.476 |
| Drugs for suppressing inflammation (only glucocorticoids or steroids) | 339 (3.1) | 1,175 (2.9) | 0.284 |
| Previous fall(s) | 740 (6.7) | 2,503 (6.2) | 0.027 |
| Physical inactivity, n (%) | 1,127 (10.3) | 3,039 (7.5) | < 0.001 |
| Lives alone, n (%) | 2,108 (19.2) | 8,379 (20.6) | 0.001 |
| Poor vision, n (%) | 2,629 (23.9) | 9,825 (24.2) | 0.594 |
| Poor hearing, n (%) | 1,992 (18.1) | 7,434 (18.3) | 0.700 |
| Maximum handgrip strength (Kg/m2), mean (SD) | 33.7 (12.0) | 34.0 (11.7) | 0.053 |

Abbreviations: n: number; BMI: body mass index; SD: standard deviation. P-values were computed by Pearson’s chi-square for categorical variables and by t-test for the continuous variable age. *non-mutually exclusive.

**Supplementary Table 2 Characteristics of study population at baseline stratified by sex**

|  | **Men**  **(n = 18,150)** | **Women**  **(n = 22,486)** | **P value** |
| --- | --- | --- | --- |
| Age (years), mean (SD) | 66.1 (9.1) | 65.6 (9.4) | < 0.001 |
| Intensity of pain, n (%):  No pain  Mild pain  Moderate pain  Severe pain | 11,827 (65.2)  1,750 (9.6)  3,417 (18.8)  1,156 (6.4) | 12,105 (53.8)  2,260 (10.1)  5,832 (25.9)  2,289 (10.2) | < 0.001 |
| N of pain sites, n (%):  No pain  1 site  ≥ 2 sites or all over | 11,827 (65.2)  3,613 (19.9)  2,710 (14.9) | 12,105 (53.8)  4,812 (21.4)  5,569 (24.8) | < 0.001 |
| Pain at specific site*, n (%):  Back  Hips  Knees  Other joints  Mouth / teeth  Other, not joint  All over | 3,257 (17.9)  1,140 (6.3)  2,114 (11.6)  1,830 (10.1)  113 (0.6)  1,534 (8.5)  231 (1.3) | 5,470 (24.3)  2,286 (10.2)  3,941 (17.5)  3,566 (15.9)  244 (1.1)  2,637 (11.7)  670 (3.0) | < 0.001  < 0.001  < 0.001  < 0.001  < 0.001  < 0.001  < 0.001 |
| Self-rated health, n (%):  Good  Poor | 12,414 (68.4)  5,736 (31.6) | 14,561 (64.8)  7,925 (35.2) | < 0.001 |
| BMI category, n (%):  Underweight  Normal  Overweight  Obese | 66 (0.4)  5,508 (30.3)  8,770 (48.3)  3,806 (21.0) | 365 (1.6)  9,042 (40.2)  8,114 (36.1)  4,965 (22.1) | < 0.001 |
| Co-morbidities, n (%): |  |  |  |
| Heart attack | 2,375 (13.1) | 1,897 (8.4) | < 0.001 |
| Hypertension | 7,185 (39.6) | 8,942 (39.8) | 0.712 |
| High cholesterol | 4,261 (23.5) | 5,348 (23.8) | 0.469 |
| Stroke | 673 (3.7) | 625 (2.8) | < 0.001 |
| Diabetes | 2,489 (13.7) | 2,435 (10.8) | < 0.001 |
| Chronic lung disease | 1,078 (5.9) | 1,207 (5.4) | 0.013 |
| Cancer | 896 (4.9) | 1,170 (5.2) | 0.224 |
| Parkinson’s disease | 128 (0.7) | 102 (0.5) | 0.001 |
| Cataracts | 1,302 (7.2) | 2,022 (9.0) | < 0.001 |
| Hip fracture | 271 (1.5) | 349 (1.6) | 0.630 |
| Other fracture | 1,049 95.8) | 1,360 (6.0) | 0.254 |
| Cognitive decline | 97 (0.5) | 117 (0.5) | 0.845 |
| Affective / emotional | 604 (3.3) | 1,581 (7.0) | < 0.001 |
| Rheumatoid arthritis | 1,037 (5.7) | 2,426 (10.8) | < 0.001 |
| Osteoarthritis / other rheumatism | 2,638 (13.0) | 4,916 (21.9) | < 0.001 |
| Drugs, n (%) |  |  |  |
| Anti-hypertensives | 7,727 (42.6) | 9,360 (41.6) | 0.055 |
| Drugs for joint pain | 2,052 (11.3) | 4,263 (19.0) | < 0.001 |
| Drugs for other pain | 1,413 (7.8) | 3,126 (13.9) | < 0.001 |
| Drugs for sleep | 821 (4.5) | 2,097 (9.3) | < 0.001 |
| Drugs for anxiety or depression | 643 (3.5) | 1,769 (7.9) | < 0.001 |
| Drugs for suppressing inflammation (only glucocorticoids or steroids) | 392 (2.2) | 783 (3.5) | < 0.001 |
| Previous fall(s) | 777 (4.3) | 1,726 (7.7) | < 0.001 |
| Lives alone, n (%) | 2,540 (14.0) | 5,839 (26.0) | < 0.001 |
| Poor vision, n (%) | 4,111 (22.7) | 5,714 (25.4) | < 0.001 |
| Poor hearing, n (%) | 3,982 (21.9) | 3,452 (15.4) | < 0.001 |
| Maximum handgrip strength (Kg/m2), mean (SD) | 43.3 (9.8) | 26.5 (6.7) | < 0.001 |
| Handgrip strength tertile**, n (%):  Low  Middle  High | 5,997 (33.0)  6,057 (33.4)  6,096 (33.6) | 6,991 (31.1)  7,901 (35.1)  7,594 (33.8) | < 0.001 |

Abbreviations: n: number; BMI: body mass index; SD: standard deviation. P-values were computed by Pearson’s chi-square for categorical variables and by t-test for continuous variables. * non-mutually exclusive; ** sex-specific.

**Supplementary Table 3 Characteristics of study population at baseline stratified by age**

|  | **Aged 50 to 64 years**  **(n = 19,542)** | **Aged 65 to 79 years**  **(n = 17,445)** | **Aged ≥ 80 years**  **(n = 3,649)** | **P value** |
| --- | --- | --- | --- | --- |
| Age (years), mean (SD) | 57.9 (4.0) | 71.0 (4.2) | 83.5 (3.2) | < 0.001 |
| Women, n (%) | 11,102 (56.8) | 9,361 (53.7) | 2,023 (55.4) | < 0.001 |
| Intensity of pain, n (%):  No pain  Mild pain  Moderate pain  Severe pain | 12,114 (62.0)  1,958 (10.0)  3,977 (20.4)  1,493 (7.6) | 10,005 (57.4)  1,738 (10.0)  4,172 (23.9)  1,530 (8.8) | 1,813 (49.7)  314 (8.6)  1,100 (30.1)  422 (11.6) | < 0.001 |
| N of pain sites, n (%):  No pain  1 site  ≥ 2 sites or all over | 12,114 (62.0)  3,894 (19.9)  3,534 (18.1) | 10,005 (57.4)  3,698 (21.2)  3,742 (21.5) | 1,813 (49.7)  833 (22.8)  1,003 (27.5) | < 0.001 |
| Pain at specific site*, n (%):  Back  Hips  Knees  Other joints  Mouth / teeth  Other, not joint  All over | 3,973 (20.3)  1,309 (6.7)  2,354 (12.0)  2,398 (12.3)  166 (0.8)  1,939 (9.9)  398 (2.0) | 3,821 (21.9)  1,679 (9.6)  2,902 (16.6)  2,413 (13.8)  163 (0.9)  1,752 (10.0)  390 (2.2) | 933 (25.6)  438 (12.0)  799 (21.9)  585 (16.0)  28 (0.8)  480 (13.2)  113 (3.1) | < 0.001  < 0.001  < 0.001  < 0.001  0.514  < 0.001  < 0.001 |
| Self-rated health, n (%):  Good  Poor | 14,387 (73.6)  5,155 (26.4) | 10,804 (61.9)  6,641 (38.1) | 1,784 (48.9)  1,865 (51.1) | < 0.001 |
| BMI category, n (%):  Underweight  Normal  Overweight  Obese | 210 (1.1)  7,289 (37.3)  7,796 (39.9)  4,247 (21.7) | 158 (0.9)  5,792 (33.2)  7,543 (43.2)  3,952 (22.7) | 63 (1.7)  1,469 (40.3)  1,545 (42.3)  572 (15.7) | < 0.001 |
| Co-morbidities, n (%): |  |  |  |  |
| Heart attack | 1,111 (5.7) | 2,341 (13.4) | 820 (22.5) | < 0.001 |
| Hypertension | 5,980 (30.6) | 8,239 (47.2) | 1,908 (52.3) | < 0.001 |
| High cholesterol | 3,918 (20.0) | 4,813 (27.6) | 878 (24.1) | < 0.001 |
| Stroke | 381 (1.9) | 681 (3.9) | 236 (6.5) | < 0.001 |
| Diabetes | 1,702 (8.7) | 2,690 (15.4) | 532 (14.6) | < 0.001 |
| Chronic lung disease | 906 (4.6) | 1,116 (6.4) | 263 (7.2) | < 0.001 |
| Cancer | 736 (3.8) | 1,098 (6.3) | 232 (6.4) | < 0.001 |
| Parkinson’s disease | 41 (0.2) | 139 (0.8) | 50 (1.4) | < 0.001 |
| Cataracts | 519 (2.7) | 1,998 (11.5) | 807 (22.1) | < 0.001 |
| Hip fracture | 137 (0.7) | 321 (1.8) | 162 (4.4) | < 0.001 |
| Other fracture | 1,107 (5.7) | 1,044 (6.0) | 258 (7.1) | 0.004 |
| Cognitive decline | 41 (0.2) | 106 (0.6) | 67 (1.8) | < 0.001 |
| Affective / emotional | 1,192 (6.1) | 814 (4.7) | 179 (4.9) | < 0.001 |
| Rheumatoid arthritis | 1,233 (6.3) | 1,755 (10.1) | 475 (13.0) | < 0.001 |
| Osteoarthritis / other rheumatism | 2,856 (14.6) | 3,513 (20.1) | 915 (25.1) | < 0.001 |
| Drugs, n (%) |  |  |  |  |
| Anti-hypertensives | 5,976 (30.6) | 8,914 (51.1) | 2,197 (60.2) | < 0.001 |
| Drugs for joint pain | 2,472 (12.6) | 3,010 (17.3) | 833 (22.8) | < 0.001 |
| Drugs for other pain | 2,079 (10.6) | 1,889 (10.8) | 571 (15.6) | < 0.001 |
| Drugs for sleep | 1,062 (5.4) | 1,370 (7.9) | 486 (13.3) | < 0.001 |
| Drugs for anxiety or depression | 1,284 (6.6) | 920 (5.3) | 208 (5.7) | < 0.001 |
| Drugs for suppressing inflammation (only glucocorticoids or steroids) | 476 (2.4) | 567 (3.3) | 132 (3.6) | < 0.001 |
| Previous fall(s) | 756 (3.9) | 1,226 (7.0) | 521 (14.3) | < 0.001 |
| Physical inactivity, n (%) | 945 (4.8) | 1,359 (7.8) | 735 (20.1) | < 0.001 |
| Lives alone, n (%) | 2,783 (14.2) | 4,025 (23.1) | 1,571 (43.1) | < 0.001 |
| Poor vision, n (%) | 4,320 (22.1) | 4,230 (24.2) | 1,275 (34.9) | < 0.001 |
| Poor hearing, n (%) | 2,355 (12.1) | 3,740 (21.4) | 1,339 (36.7) | < 0.001 |
| Maximum handgrip strength (Kg/m2), mean (SD) | 36.9 (11.9) | 32.5 (10.9) | 25.7 (9.2) | < 0.001 |
| Handgrip strength tertile**, n (%):  Low  Middle  High | 3,409 (17.4)  6,509 (33.3)  9,624 (49.2) | 6,806 (39.0)  6,709 (38.5)  3,930 (22.5) | 2,773 (76.0)  740 (20.3)  136 (3.7) | < 0.001 |

Abbreviations: n: number; BMI: body mass index; SD: standard deviation. P-values were computed by Pearson’s chi-square for categorical variables and by analysis of variance (ANOVA) for continuous variables. *non-mutually exclusive; **sex-specific.

**Supplementary Table 4 Intensity of pain in men at baseline, by country (n = 18,150 men)**

|  | **No pain**  **(n = 11,827)** | **Mild pain**  **(n = 1,750)** | **Moderate pain**  **(n = 3,417)** | **Severe pain**  **(n = 1,156)** | **P value** |
| --- | --- | --- | --- | --- | --- |
| Austria, n = 1,089 | 749 (68.8) | 68 (6.2) | 231 (21.2) | 41 (3.8) | < 0.001 |
| Germany, n = 1,897 | 1,299 (68.5) | 85 (4.5) | 345 (18.2) | 168 (8.9) |  |
| Sweden, n = 1,531 | 1,103 (72.0) | 140 (9.1) | 228 (14.9) | 60 (3.9) |  |
| Spain, n = 1,909 | 1,344 (70.4) | 154 (8.1) | 308 (16.1) | 103 (5.4) |  |
| Italy, n = 1,401 | 820 (58.5) | 208 (14.8) | 284 (20.3) | 89 (6.4) |  |
| France, n = 1,254 | 617 (49.2) | 163 (13.0) | 314 (25.0) | 160 (12.8) |  |
| Denmark, n = 1,429 | 1,073 (75.1) | 112 (7.8) | 191 (13.4) | 53 (3.7) |  |
| Switzerland, n = 1,109 | 888 (80.1) | 69 (6.2) | 121 (10.9) | 31 (2.8) |  |
| Belgium, n = 1,741 | 1,145 (65.8) | 202 (11.6) | 274 (15.7) | 120 (6.9) |  |
| Israel, n = 495 | 338 (68.3) | 46 (9.3) | 85 (17.2) | 26 (5.3) |  |
| Czech Republic, n = 1,531 | 907 (59.2) | 219 (14.3) | 321 (21.0) | 84 (5.5) |  |
| Luxembourg, n = 449 | 285 (63.5) | 53 (11.8) | 75 (16.7) | 36 (8.0) |  |
| Slovenia, n = 892 | 489 (54.8) | 67 (7.5) | 265 (29.7) | 71 (8.0) |  |
| Estonia, n = 1,423 | 770 (54.1) | 164 (11.5) | 375 (26.4) | 114 (8.0) |  |

Abbreviation: n: number. P-value was computed by Pearson’s chi-square.

**Supplementary Table 5 Intensity of pain in women at baseline, by country (n = 22,486 women)**

|  | **No pain**  **(n = 12,105)** | **Mild pain**  **(n = 2,260)** | **Moderate pain**  **(n = 5,832)** | **Severe pain**  **(n = 2,289)** | **P value** |
| --- | --- | --- | --- | --- | --- |
| Austria, n = 1,500 | 930 (62.0) | 97 (6.5) | 357 (23.8) | 116 (7.7) | < 0.001 |
| Germany, n = 2,011 | 1,186 (59.0) | 97 (4.8) | 468 (23.3) | 260 (12.9) |  |
| Sweden, n = 1,735 | 1,005 (57.9) | 222 (12.8) | 402 (23.2) | 106 (6.1) |  |
| Spain, n = 2,097 | 1,061 (50.6) | 204 (9.7) | 564 (26.9) | 268 (12.8) |  |
| Italy, n = 1,626 | 637 (39.2) | 292 (18.0) | 524 (32.2) | 173 (10.6) |  |
| France, n = 1,636 | 638 (39.0) | 196 (12.0) | 501 (30.6) | 301 (18.4) |  |
| Denmark, n = 1,645 | 1,075 (65.3) | 143 (8.7) | 305 (18.5) | 122 (7.4) |  |
| Switzerland, n = 1,271 | 902 (71.0) | 76 (6.0) | 227 (17.9) | 66 (5.2) |  |
| Belgium, n = 2,066 | 1,155 (55.9) | 219 (10.6) | 496 (24.0) | 196 (9.5) |  |
| Israel, n = 613 | 347 (56.6) | 54 (8.8) | 152 (24.8) | 60 (9.8) |  |
| Czech Republic, n = 2,225 | 1,146 (51.5) | 315 (14.2) | 595 (26.7) | 169 (7.6) |  |
| Luxembourg, n = 504 | 287 (56.9) | 29 (5.8) | 127 (25.2) | 61 (12.1) |  |
| Slovenia, n = 1,179 | 521 (44.2) | 104 (8.8) | 399 (33.8) | 155 (13.1) |  |
| Estonia, n = 2,378 | 1,215 (51.1) | 212 (8.9) | 715 (30.1) | 236 (9.9) |  |

Abbreviation: n: number. P-value was computed by Pearson’s chi-square.

**Supplementary Table 6 Pain characteristics in the study population at baseline, by intensity of pain**

|  | **Mild pain**  **(n = 4,010)** | **Moderate pain**  **(n = 9,249)** | **Severe pain**  **(n = 3,445)** | **P value** |
| --- | --- | --- | --- | --- |
| Pain at specific site, n (%): |  |  |  |  |
| Back | 1,824 (45.5) | 4,923 (53.2) | 1,980 (57.5) | < 0.001 |
| Hips | 572 (14.3) | 1,952 (21.1) | 902 (26.2) | < 0.001 |
| Knees | 1,184 (29.5) | 3,440 (37.2) | 1,431 (41.5) | < 0.001 |
| Other joints | 1,150 (28.7) | 2,985 (32.3) | 1,261 (36.6) | < 0.001 |
| Mouth / teeth | 60 (1.5) | 190 (2.1) | 107 (3.1) | < 0.001 |
| Other, not joints | 849 (21.2) | 2,260 (24.4) | 1,062 (30.8) | < 0.001 |
| All over | 84 (2.1) | 488 (5.3) | 329 (9.6) | < 0.001 |
| N of pain sites, n (%):  No pain  1 site  ≥ 2 sites or all over | 0 (0)  2,645 (66.0)  1,365 (34.0) | 0 (0)  4,486 (48.5)  4,763 (51.5) | 0 (0)  1,294 (37.6)  2,151 (62.4) | < 0.001 |
| Joint pain in previous six months, n (%):  No  Yes  Not applicable | 410 (10.2)  2,985 (74.5)  615 (15.3) | 453 (4.9)  7,424 (80.3)  1,372 (14.8) | 95 (2.8)  2,740 (79.5)  610 (17.7) | < 0.001 |

Abbreviation: n: number, SD: standard deviation. P-values were computed by Pearson’s chi-square for categorical variables.

**Supplementary Table 7 History of falls at baseline, by sex and country**

|  | **n of men** | **n (%) of men**  **with history of falls** | **P**  **value*** | **n of women** | **n (%) of women**  **with history of falls** | **P value**** |
| --- | --- | --- | --- | --- | --- | --- |
| All countries | 18,150 | 777 (4.3) | < 0.001 | 22,486 | 1,726 (7.7) | < 0.001 |
| Austria | 1,089 | 36 (3.3) |  | 1,500 | 86 (5.7) |  |
| Germany | 1,897 | 76 (4.0) |  | 2,011 | 122 (6.1) |  |
| Sweden | 1,531 | 56 (3.7) |  | 1,735 | 108 (6.2) |  |
| Spain | 1,909 | 93 (4.9) |  | 2,097 | 212 (10.1) |  |
| Italy | 1,401 | 39 (2.8) |  | 1,626 | 89 (5.5) |  |
| France | 1,254 | 70 (5.6) |  | 1,636 | 188 (11.5) |  |
| Denmark | 1,429 | 40 (2.8) |  | 1,645 | 62 (3.8) |  |
| Switzerland | 1,109 | 32 (2.9) |  | 1,271 | 65 (5.1) |  |
| Belgium | 1,741 | 83 (4.8) |  | 2,066 | 180 (8.7) |  |
| Israel | 495 | 21 (4.2) |  | 613 | 47 (7.7) |  |
| Czech Republic | 1,531 | 94 (6.1) |  | 2,225 | 239 (10.7) |  |
| Luxembourg | 449 | 23 (5.1) |  | 504 | 67 (13.3) |  |
| Slovenia | 892 | 28 (3.1) |  | 1,179 | 58 (4.9) |  |
| Estonia | 1,423 | 86 (6.0) |  | 2,378 | 203 (8.5) |  |

Abbreviation: n: number. P-value* and P-value** for the distribution of history of falls by country in men and women, respectively, were computed by Pearson’s chi-square.

**Supplementary Table 8 Cross-sectional association between intensity of pain and falls risk at baseline**

|  | **All**  **(n = 40,636)** | | | **Men**  **(n = 18,150)** | | | **Women**  **(n = 22,486)** | | |
| --- | --- | --- | --- | --- | --- | --- | --- | --- | --- |
|  | **n of falls** | **OR [95% CI]** | **P value** | **n of falls** | **OR [95% CI]** | **P value** | **n of falls** | **OR [95% CI]** | **P value** |
| **Model 1** |  |  |  |  |  |  |  |  |  |
| No pain | 799 | 1 (ref) |  | 308 | 1 (ref) |  | 491 | 1 (ref) |  |
| Mild pain | 231 | 1.69 [1.45; 1.97] | < 0.001 | 79 | 1.76 [1.37; 2.27] | < 0.001 | 152 | 1.65 [1.37; 2.00] | < 0.001 |
| Moderate pain | 897 | 2.68 [2.42; 2.96] | < 0.001 | 253 | 2.80 [2.35; 3.32] | < 0.001 | 644 | 2.62 [2.32; 2.96] | < 0.001 |
| Severe pain | 576 | 5.01 [4.46; 5.63] | < 0.001 | 137 | 4.85 [3.92; 6.01] | < 0.001 | 439 | 5.05 [4.39; 5.81] | < 0.001 |
| **Model 2** |  |  |  |  |  |  |  |  |  |
| No pain | 799 | 1 (ref) |  | 308 | 1 (ref) |  | 491 | 1 (ref) |  |
| Mild pain | 231 | 1.40 [1.20; 1.64] | < 0.001 | 79 | 1.41 [1.08; 1.83] | 0.011 | 152 | 1.39 [1.14; 1.69] | 0.001 |
| Moderate pain | 897 | 1.76 [1.57; 1.97] | < 0.001 | 253 | 1.79 [1.48; 2.17] | < 0.001 | 644 | 1.74 [1.51; 1.99] | < 0.001 |
| Severe pain | 576 | 2.54 [2.21; 2.91] | < 0.001 | 137 | 2.38 [1.86; 3.05] | < 0.001 | 439 | 2.60 [2.21; 3.07] | < 0.001 |
| **Model 3** |  |  |  |  |  |  |  |  |  |
| No pain | 799 | 1 (ref) |  | 308 | 1 (ref) |  | 491 | 1 (ref) |  |
| Mild pain | 231 | 1.36 [1.16; 1.59] | < 0.001 | 79 | 1.36 [1.04; 1.77] | 0.022 | 152 | 1.34 [1.11; 1.64] | 0.003 |
| Moderate pain | 897 | 1.62 [1.44; 1.82] | < 0.001 | 253 | 1.65 [1.35; 2.01] | < 0.001 | 644 | 1.59 [1.38; 1.84] | < 0.001 |
| Severe pain | 576 | 2.21 [1.92; 2.56] | < 0.001 | 137 | 2.08 [1.60; 2.70] | < 0.001 | 439 | 2.26 [1.90; 2.70] | < 0.001 |
| **Model 4** |  |  |  |  |  |  |  |  |  |
| No pain | 799 | 1 (ref) |  | 308 | 1 (ref) |  | 491 | 1 (ref) |  |
| Mild pain | 231 | 1.36 [1.16; 1.59] | < 0.001 | 79 | 1.37 [1.05; 1.79] | 0.019 | 152 | 1.34 [1.10; 1.63] | 0.004 |
| Moderate pain | 897 | 1.59 [1.41; 1.78] | < 0.001 | 253 | 1.62 [1.32; 1.98] | < 0.001 | 644 | 1.56 [1.35; 1.80] | < 0.001 |
| Severe pain | 576 | 2.11 [1.83; 2.44] | < 0.001 | 137 | 1.97 [1.51; 2.56] | < 0.001 | 439 | 2.17 [1.82; 2.59] | < 0.001 |

Odds ratios and 95% confidence intervals were calculated by binary logistic regression. Model 1: adjusted for age and sex. Model 2: Model 1 + self-rated health, BMI category, heart attack, hypertension, high cholesterol, stroke, diabetes, chronic lung disease, cancer, Parkinson’s, cataracts, hip fracture, other fractures, cognitive impairment, affective / emotional disorder, rheumatoid arthritis, osteoarthritis or other rheumatism, poor vision, poor hearing, lives alone, country. Model 3: Model 2 + anti-hypertensives; drugs for joint pain; drugs for other pain; drugs for sleep; drugs for anxiety or depression; drugs for suppressing inflammation (only glucocorticoids or steroids). Model 4: Model 3 + physical inactivity, sex-specific tertiles of handgrip strength. Number of participants reporting fall(s) in each category: all participants: 2,503; men: 777; women: 1,726. Abbreviations: OR: odds ratios; CI: confidence intervals; ref: reference.

**Supplementary Table 9 Longitudinal association between intensity of pain and falls risk at follow-up, by three age categories**

|  | **Aged 50 to 64 years**  **(n = 19,542)** | | | **Aged 65 to 79 years**  **(n = 17,445)** | | | **Aged ≥ 80 years**  **(n = 3,649)** | | | **P*** |
| --- | --- | --- | --- | --- | --- | --- | --- | --- | --- | --- |
|  | **n of falls** | **OR [95% CI]** | **P value** | **n of falls** | **OR [95% CI]** | **P value** | **n of falls** | **OR [95% CI]** | **P value** |  |
| **Model 1** |  |  |  |  |  |  |  |  |  |  |
| No pain | 295 | 1 (ref) |  | 541 | 1 (ref) |  | 247 | 1 (ref) |  | < 0.001 |
| Mild pain | 70 | 1.44 [1.11; 1.88] | 0.007 | 131 | 1.35 [1.11; 1.65] | 0.003 | 46 | 1.09 [0.77; 1.53] | 0.632 |  |
| Moderate pain | 255 | 2.59 [2.18; 3.08] | < 0.001 | 482 | 2.06 [1.81; 2.34] | < 0.001 | 212 | 1.43 [1.16; 1.75] | 0.001 |  |
| Severe pain | 144 | 4.06 [3.30; 5.00] | < 0.001 | 272 | 3.32 [2.83; 3.89] | < 0.001 | 110 | 2.08 [1.60; 2.70] | < 0.001 |  |
| **Model 2** |  |  |  |  |  |  |  |  |  |  |
| No pain | 295 | 1 (ref) |  | 541 | 1 (ref) |  | 247 | 1 (ref) |  | < 0.001 |
| Mild pain | 70 | 1.24 [0.94; 1.62] | 0.127 | 131 | 1.14 [0.93; 1.40] | 0.217 | 46 | 0.89 [0.62; 1.27] | 0.509 |  |
| Moderate pain | 255 | 1.69 [1.39; 2.06] | < 0.001 | 482 | 1.44 [1.24; 1.67] | < 0.001 | 212 | 1.07 [0.85; 1.35] | 0.561 |  |
| Severe pain | 144 | 1.94 [1.51; 2.50] | < 0.001 | 272 | 1.74 [1.43; 2.10] | < 0.001 | 110 | 1.25 [0.92; 1.70] | 0.154 |  |
| **Model 3** |  |  |  |  |  |  |  |  |  |  |
| No pain | 295 | 1 (ref) |  | 541 | 1 (ref) |  | 247 | 1 (ref) |  | < 0.001 |
| Mild pain | 70 | 1.23 [0.94; 1.62] | 0.132 | 131 | 1.13 [0.92; 1.39] | 0.256 | 46 | 0.84 [0.58; 1.21] | 0.342 |  |
| Moderate pain | 255 | 1.68 [1.37; 2.05] | < 0.001 | 482 | 1.38 [1.19; 1.61] | < 0.001 | 212 | 0.99 [0.77; 1.26] | 0.921 |  |
| Severe pain | 144 | 1.87 [1.43; 2.43] | < 0.001 | 272 | 1.62 [1.32; 1.99] | < 0.001 | 110 | 1.11 [0.80; 1.52] | 0.538 |  |
| **Model 4** |  |  |  |  |  |  |  |  |  |  |
| No pain | 295 | 1 (ref) |  | 541 | 1 (ref) |  | 247 | 1 (ref) |  | < 0.001 |
| Mild pain | 70 | 1.24 [0.94; 1.63] | 0.125 | 131 | 1.13 [0.91; 1.39] | 0.263 | 46 | 0.83 [0.57; 1.19] | 0.308 |  |
| Moderate pain | 255 | 1.64 [1.34; 2.02] | < 0.001 | 482 | 1.36 [1.17; 1.59] | < 0.001 | 212 | 0.99 [0.77; 1.26] | 0.923 |  |
| Severe pain | 144 | 1.76 [1.34; 2.30] | < 0.001 | 272 | 1.56 [1.27; 1.92] | < 0.001 | 110 | 1.09 [0.79; 1.51] | 0.599 |  |

Odds ratios and 95% confidence intervals were calculated by binary logistic regression. Model 1: adjusted for age and sex. Model 2: Model 1 + self-rated health, BMI category, heart attack, hypertension, high cholesterol, stroke, diabetes, chronic lung disease, cancer, Parkinson’s, cataracts, hip fracture, other fractures, cognitive impairment, affective / emotional disorder, rheumatoid arthritis, osteoarthritis or other rheumatism, poor vision, poor hearing, lives alone, previous falls, country. Model 3: Model 2 + anti-hypertensives; drugs for joint pain; drugs for other pain; drugs for sleep; drugs for anxiety or depression; drugs for suppressing inflammation (only glucocorticoids or steroids). Model 4: Model 3 + physical inactivity, sex-specific tertiles of handgrip strength. Number of participants reporting fall(s) at follow-up in each category: aged 50 to 64 years: 764; aged 65 to 79 years: 1,426; aged ≥ 80 years: 615. Abbreviations: OR: odds ratios; CI: confidence intervals; ref: reference. P* is P value for interaction between age and intensity of pain; interaction term was computed by multiplying age (continuous) by intensity of pain.

**Supplementary Table 10 Longitudinal association between intensity of pain and falls risk at follow-up in participants without history of falls or hip or other fractures at baseline (n = 35,684)**

|  | **All**  **(n = 35,684)** | | | **Men**  **(n =16,233)** | | | **Women**  **(n = 19,451)** | | |
| --- | --- | --- | --- | --- | --- | --- | --- | --- | --- |
|  | **n of falls** | **OR [95% CI]** | **P value** | **n of falls** | **OR [95% CI]** | **P value** | **n of falls** | **OR [95% CI]** | **P value** |
| **Model 1** |  |  |  |  |  |  |  |  |  |
| No pain | 855 | 1 (ref) |  | 316 | 1 (ref) |  | 539 | 1 (ref) |  |
| Mild pain | 169 | 1.19 [1.00; 1.41] | 0.049 | 62 | 1.37 [1.04; 1.81] | 0.027 | 107 | 1.09 [0.88; 1.35] | 0.421 |
| Moderate pain | 623 | 1.86 [1.67; 2.07] | < 0.001 | 186 | 2.14 [1.78; 2.58] | < 0.001 | 437 | 1.74 [1.52; 1.98] | < 0.001 |
| Severe pain | 273 | 2.57 [2.22; 2.97] | < 0.001 | 61 | 2.36 [1.77; 3.14] | < 0.001 | 212 | 2.61 [2.20; 3.10] | < 0.001 |
| **Model 2** |  |  |  |  |  |  |  |  |  |
| No pain | 855 | 1 (ref) |  | 316 | 1 (ref) |  | 539 | 1 (ref) |  |
| Mild pain | 169 | 1.05 [0.88; 1.25] | 0.602 | 62 | 1.20 [0.90; 1.60] | 0.207 | 107 | 0.96 [0.77; 1.19] | 0.702 |
| Moderate pain | 623 | 1.43 [1.27; 1.62] | < 0.001 | 186 | 1.66 [1.34; 2.05] | < 0.001 | 437 | 1.33 [1.14; 1.54] | < 0.001 |
| Severe pain | 273 | 1.67 [1.42; 1.98] | < 0.001 | 61 | 1.51 [1.09; 2.08] | 0.012 | 212 | 1.70 [1.39; 2.07] | < 0.001 |
| **Model 3** |  |  |  |  |  |  |  |  |  |
| No pain | 855 | 1 (ref) |  | 316 | 1 (ref) |  | 539 | 1 (ref) |  |
| Mild pain | 169 | 1.03 [0.86; 1.23] | 0.741 | 62 | 1.18 [0.88; 1.57] | 0.266 | 107 | 0.94 [0.76; 1.18] | 0.604 |
| Moderate pain | 623 | 1.37 [1.21; 1.55] | < 0.001 | 186 | 1.57 [1.26; 1.96] | < 0.001 | 437 | 1.27 [1.09; 1.49] | 0.002 |
| Severe pain | 273 | 1.54 [1.29; 1.83] | < 0.001 | 61 | 1.37 [0.98; 1.92] | 0.066 | 212 | 1.56 [1.27; 1.93] | < 0.001 |
| **Model 4** |  |  |  |  |  |  |  |  |  |
| No pain | 855 | 1 (ref) |  | 316 | 1 (ref) |  | 539 | 1 (ref) |  |
| Mild pain | 169 | 1.03 [0.86; 1.23] | 0.735 | 62 | 1.18 [0.89; 1.58] | 0.252 | 107 | 0.94 [0.75; 1.17] | 0.579 |
| Moderate pain | 623 | 1.35 [1.19; 1.53] | < 0.001 | 186 | 1.57 [1.26; 1.95] | < 0.001 | 437 | 1.25 [1.07; 1.46] | 0.005 |
| Severe pain | 273 | 1.47 [1.23; 1.75] | < 0.001 | 61 | 1.30 [0.92; 1.82] | 0.136 | 212 | 1.49 [1.21; 1.84] | < 0.001 |

Odds ratios and 95% confidence intervals were calculated by binary logistic regression. Model 1: adjusted for age and sex. Model 2: Model 1 + self-rated health, BMI category, heart attack, hypertension, high cholesterol, stroke, diabetes, chronic lung disease, cancer, Parkinson’s, cataracts, cognitive impairment, affective / emotional disorder, rheumatoid arthritis, osteoarthritis or other rheumatism, poor vision, poor hearing, lives alone, previous falls, country. Model 3: Model 2 + anti-hypertensives; drugs for joint pain; drugs for other pain; drugs for sleep; drugs for anxiety or depression; drugs for suppressing inflammation (only glucocorticoids or steroids). Model 4: Model 3 + physical inactivity, sex-specific tertiles of handgrip strength. Number of participants reporting fall(s) at follow-up in each category: all participants: 1,920; men: 625; women: 1,295. Abbreviations: OR: odds ratios; CI: confidence intervals; ref: reference.

**Supplementary Table 11 Longitudinal association between number of pain sites and falls risk at follow-up in 16,704 participants with pain at baseline**

|  | **All**  **(n = 16,704)** | | | **Men**  **(n = 6,323)** | | | **Women**  **(n = 10,381)** | | |
| --- | --- | --- | --- | --- | --- | --- | --- | --- | --- |
|  | **n of falls** | **OR [95% CI]** | **P value** | **n of falls** | **OR [95% CI]** | **P value** | **n of falls** | **OR [95% CI]** | **P value** |
| **Model 1** |  |  |  |  |  |  |  |  |  |
| 1 site | 628 | 1 (ref) |  | 195 | 1 (ref) |  | 433 | 1 (ref) |  |
| ≥ 2 sites or all over | 1,094 | 1.75 [1.57; 1.94] | < 0.001 | 297 | 2.07 [1.72; 2.51] | < 0.001 | 797 | 1.62 [1.43; 1.84] | < 0.001 |
| **Model 2** |  |  |  |  |  |  |  |  |  |
| 1 site | 628 | 1 (ref) |  | 195 | 1 (ref) |  | 433 | 1 (ref) |  |
| ≥ 2 sites or all over | 1,094 | 1.36 [1.21; 1.52] | < 0.001 | 297 | 1.56 [1.26; 1.92] | < 0.001 | 797 | 1.28 [1.11; 1.46] | < 0.001 |
| **Model 3** |  |  |  |  |  |  |  |  |  |
| 1 site | 628 | 1 (ref) |  | 195 | 1 (ref) |  | 433 | 1 (ref) |  |
| ≥ 2 sites or all over | 1,094 | 1.30 [1.16; 1.47] | < 0.001 | 297 | 1.52 [1.23; 1.88] | < 0.001 | 797 | 1.22 [1.06; 1.40] | 0.006 |
| **Model 4** |  |  |  |  |  |  |  |  |  |
| 1 site | 628 | 1 (ref) |  | 195 | 1 (ref) |  | 433 | 1 (ref) |  |
| ≥ 2 sites or all over | 1,094 | 1.29 [1.14; 1.45] | < 0.001 | 297 | 1.51 [1.22; 1.87] | < 0.001 | 797 | 1.20 [1.04; 1.38] | 0.012 |

Odds ratios and 95% confidence intervals were calculated by binary logistic regression. Model 1: adjusted for age and sex. Model 2: Model 1 + self-rated health, BMI category, heart attack, hypertension, high cholesterol, stroke, diabetes, chronic lung disease, cancer, Parkinson’s, cataracts, hip fracture, other fractures, cognitive impairment, affective / emotional disorder, rheumatoid arthritis, osteoarthritis or other rheumatism, poor vision, poor hearing, lives alone, previous falls, country. Model 3: Model 2 + anti-hypertensives; drugs for joint pain; drugs for other pain; drugs for sleep; drugs for anxiety or depression; drugs for suppressing inflammation (only glucocorticoids or steroids). Model 4: Model 3 + physical inactivity, sex-specific tertiles of handgrip strength. Number of participants reporting fall(s) at follow-up in each category: all participants: 1,722; men: 492; women: 1,230. Abbreviations: OR: odds ratios; CI: confidence intervals; ref: reference.
